# Traction-assisted endoscopic submucosal dissection of a neuroendocrine tumor in the gastric body of a patient with autoimmune gastritis

**DOI:** 10.1055/a-2738-6905

**Published:** 2025-11-19

**Authors:** Angelo Bruni, Liboria Laterza, Francesco Bombaci, Michele Dota, Rosario Arena, Giovanni Barbara, Paolo Cecinato

**Affiliations:** 1 198207 Department of Medical and Surgical Sciences, University of Bologna, Bologna, Italy; 2 18508 Gastroenterology Unit, IRCCS Azienda Ospedaliero-Universitaria di Bologna, Bologna, Italy; 3 19001 Department of Internal Medicine and Medical Therapy, University of Pavia, Pavia, Italy; 4 18560 Department of Gastroenterology and GI Endoscopy, University Hospital Arcispedale Sant'Anna of Ferrara, Ferrara, Italy

A 75-year-old woman with known autoimmune atrophic gastritis (AAG) and previous resections of millimetric neuroendocrine tumors (NETs) presented for evaluation of a newly detected 25 × 20-mm lesion on the greater curvature of the gastric body. Vitamin B12 deficiency had also been documented in the context of AAG 1 2 .

A previous esophagogastroduodenoscopy (EGDS) confirmed the presence of a 25 × 20 mm suspected NET on the greater curvature of the gastric body. The lesion was marked, and an endoscopic submucosal dissection (ESD) was performed using a J-type hook-knife (Olympus Co. Ltd, Tokyo, Japan; Fig. 1 ). Traction was achieved through a double clip-and-band technique, which allowed for precise dissection of the lesion without significant intra-procedural bleeding ( Fig. 2 **a** ; Video 1 ). The lesion was resected en bloc, and the resection site was closed with clips to ensure hemostasis ( Fig. 2 **b** ).

**Fig. 1:**
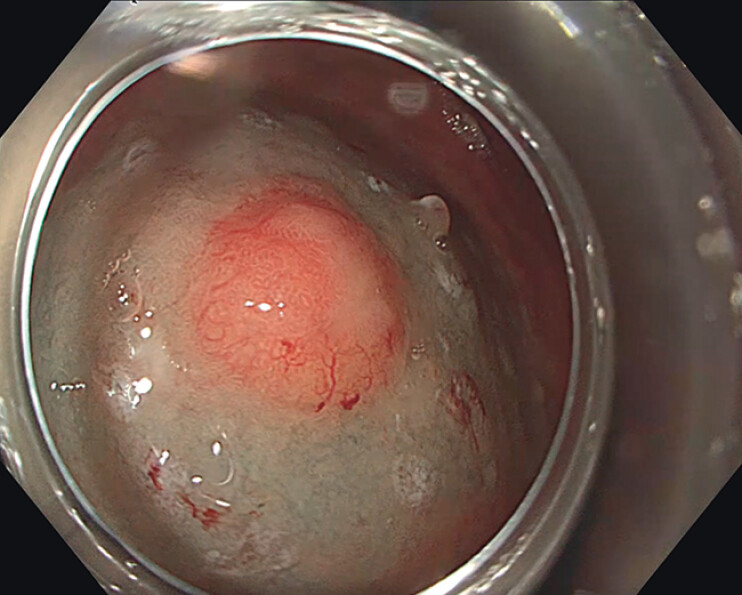
Endoscopic appearance of the gastric neuroendocrine tumor. A subepithelial lesion on the greater curvature of the gastric body, identified in a patient with autoimmune atrophic gastritis.

**Fig. 2:**
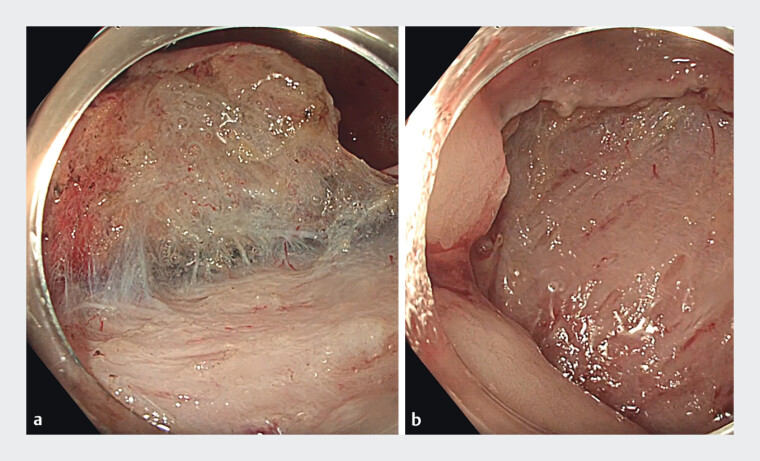
Traction-assisted endoscopic submucosal dissection of the gastric NET. **a** The lesion is partially dissected, revealing the submucosal plane with visible fibrotic strands, facilitated by the double clip-and-band traction technique. **b** Post-resection appearance of the gastric muscular layer after en-bloc excision.

Traction-assisted endoscopic submucosal dissection (ESD) of a 25 mm × 20 mm gastric neuroendocrine tumor (NET) using a double clip-and-band technique in a patient with autoimmune atrophic gastritis.Video 1

Histopathology confirmed a R0 resection of a well-differentiated NET G1 (synaptophysin+, INSM1+, and gastrin-negative), which infiltrated the submucosa. Immunohistochemistry revealed strong expression of somatostatin receptor 2A (SSTR2A, >95% membranous positivity) and weaker expression of SSTR5. The Ki-67 proliferation index was 2.9%, indicating low proliferative activity, without peritumoral lymphatic involvement.

A follow-up EGDS demonstrated a well-healed resection scar on the greater curvature with no signs of recurrence upon high-definition white light and narrow band imaging (Olympus Co. Ltd, Tokyo, Japan) evaluation. However, five new small lesions suspicious for NETs were identified in the body/fundus and deemed suitable for endoscopic resection, highlighting the propensity for multifocal NETs in AAG.

This video case underscores the feasibility of traction-assisted ESD for large gastric NETs in AAG 3 4 . By providing stable exposure of the submucosal plane, traction can enable precise dissection and secure margin clearance while preserving muscular integrity. Such an approach may be particularly valuable in patients with chronic inflammatory conditions that predispose them to multiple or recurrent gastric lesions 5 .

Endoscopy_UCTN_Code_TTT_1AO_2AG_3AD

## Notes

Endoscopy E-Videos https://eref.thieme.de/e-videos
*E-Videos* is an open access online section of the journal *Endoscopy* , reporting on interesting cases and new techniques in gastroenterological endoscopy. All papers include a high-quality video and are published with a Creative Commons CC-BY license. Endoscopy E-Videos qualify for HINARI discounts and waivers and eligibility is automatically checked during the submission process. We grant 100% waivers to articles whose corresponding authors are based in Group A countries and 50% waivers to those who are based in Group B countries as classified by Research4Life (see: https://www.research4life.org/access/eligibility/ ). This section has its own submission website at https://mc.manuscriptcentral.com/e-videos .

## References

[1] Lamberti G Panzuto F Pavel M Gastric neuroendocrine neoplasms Nat Rev Dis Primers 2024 10 1 17 10.1038/s41572-024-00508-y 38605021

[2] Rindi G Mete O Uccella S Overview of the 2022 WHO classification of neuroendocrine neoplasms Endocr Pathol 2022 33 115 154 10.1007/s12022-022-09708-2 35294740

[3] Pimentel-Nunes P Libânio D Bastiaansen BAJ Endoscopic submucosal dissection for superficial gastrointestinal lesions: European Society of Gastrointestinal Endoscopy (ESGE) Guideline – Update 2022 Endoscopy 2022 54 591 622 10.1055/A-1811-7025 35523224

[4] Panzuto F Parodi MC Esposito G Endoscopic management of gastric, duodenal and rectal NETs: Position paper from the Italian Association for Neuroendocrine Tumors (Itanet), Italian Society of Gastroenterology (SIGE), Italian Society of Digestive Endoscopy (SIED) Dig Liver Dis 2024 56 589 600 10.1016/J.DLD.2023.12.015 38216439

[5] Noh JH Kim DH Yoon H Clinical Outcomes of Endoscopic Treatment for Type 1 Gastric Neuroendocrine Tumor J Gastrointest Surg 2021 25 2495 2502 10.1007/S11605-021-04997-0 33825119

